# Comparison between two fast threshold strategies: SPARK and SITA in normal subjects

**DOI:** 10.1177/1120672120926455

**Published:** 2020-05-29

**Authors:** Say Kiang Foo, Robert Peter Cubbidge, Rebekka Heitmar

**Affiliations:** 1 SEGi University, Faculty of Optometry & Vision Sciences, Petaling Jaya, Malaysia; 2 School of Life and Health Sciences, Aston University, Birmingham, UK

**Keywords:** Clinical tests, glaucoma, lens, cataract, optics, refraction, instruments, psychophysical testing, retina, techniques of retinal examination

## Abstract

**Background:**

Numerous fast threshold strategies have been developed in perimetry which use maximum likelihood approaches to estimate the threshold. A recent approach to threshold estimation has been developed estimating the threshold from a limited number of test points which further reduces examination time. This strategy, SPARK, has not been compared to the SITA strategy. The aim of this study was to compare SPARK with SITA in a normal cohort to evaluate within and between strategy agreement in threshold estimates.

**Methods:**

A total of 83 normal subjects each underwent two visual field examinations with SITA and SPARK on two separate occasions on a randomly selected eye. The eye examined and the order of strategy examined first was randomised but remained constant over the two perimetry visits.

**Results:**

Visual field examination with SPARK Precision was on average 33% faster than SITA Standard. A positive correlation between group mean sensitivities of SITA Standard and SPARK Precision (rho = 0.713, p < 0.001) was found. In total, 95% of stimulus locations were located within the 95% limits of agreement and linear regression on the differences in sensitivities showed no statistically significant proportional bias (t = 1.713, p = 0.09). Pointwise analysis showed SITA Standard had significantly larger variability for individual stimulus locations examined over two visits when compared to SPARK (t = 9.175, p < 0.001).

**Conclusion:**

The clinical examination of SPARK yields a sensitivity profile similar to SITA but in a faster examination time. The lower threshold variability of SPARK may be as a result of data smoothing in the threshold estimation process.

## Background

Standard automated perimetry (SAP) remains an essential clinical assessment of visual function in patients suffering from glaucoma.^1^ A number of threshold estimation algorithms have been developed to achieve accurate estimation of the differential light threshold in an acceptable test time, thereby reducing the influence of fatigue^2–9^ on the results. Such algorithms exhibit greater efficiency in threshold estimation since they are based on maximum likelihood theory which results in fewer stimulus presentations compared with predecessor algorithms. The SITA algorithm used in the Humphrey Field Analyser can be considered as a ‘gold standard’ reference to other perimeters as extensive visual field research has been carried out using this instrumentation. Threshold variability defined by both short-term and long-term fluctuations can influence the statistical and clinical analysis of visual fields, particularly in measures of focal visual field loss.

A different approach to achieving efficient threshold estimation has been developed by de la Rosa and Gonzalez-Hernandez.^10^ The SPARK Precision algorithm is a fast threshold strategy intended to use the information from a limited number of test points to estimate threshold values in the central visual field within a short duration of examination. It uses the statistical relationships between neighbouring test points of the visual field which are in sectors which are thought to contain the same ganglion cell fibre bundles which correspond to morphologically and functionally defined regions.^11,12^ In brief, the model was derived from a sample of more than 90,000 visual field examinations (originating from 14,923 patients) obtained using the G1 program (TOP strategy, Octopus 1-2-3 perimeter). Four threshold estimates are determined in four different phases, and a final threshold estimate is then averaged for each testing point.^10^ By averaging the thresholds, a reduction in threshold fluctuation of approximately 40% has been reported in patients in the early stages of glaucoma^13^ and test durations of less than 3 min have been achieved.^14^ The initial phase of the SPARK Precision algorithm (also referred to as SPARK training) can be completed in less than 40 s^15^ and SPARK Precision has exhibited good diagnostic sensitivity and specificity in glaucoma patients even when utilising the first phase results only.^15^ Moreover, the agreement of SPARK Precision with morphologic indices^15^ enabled its threshold sensitivity to be used to predict the thickness of retinal nerve fibre layer.^16^ The objective of this study was to evaluate the mean sensitivity (MS) and pointwise analysis of threshold values of SPARK Precision in comparison with the Humphrey Field Analyser in a normal population.

## Methods

A total of 83 normal subjects (49 males; mean age: 39.6 years; standard deviation (SD): 16.1; range: 20–71) were recruited from the optometry clinics at SEGi University, Malaysia. Inclusion criteria were absence of any ocular disease; any impairment of the transparency of ocular media; intraocular pressure (IOP) ≤21 mmHg; a previously normal visual field; normal optic disc appearance, that is, no localised neuroretinal rim loss; an absence of optic disc haemorrhage, notches or pallor; a cup/disc ratio ≤0.6 or cup/disc asymmetry ≤0.2; and no nerve fibre layer defects or a positive family history of glaucoma. Additional inclusion criteria were best-corrected visual acuity (BCVA) of 6/6 or better; refractive errors below ±6.00-DS spherical error and less than 2.50-DC astigmatism; no history of intraocular surgery or other ocular diseases that could affect the visual field. All participants were free from any systemic disease that could affect ocular health such as diabetes mellitus (DM) and hypertension (HT). Participants were asked to refrain from caffeine, alcohol and nicotine use for a minimum of 2 h prior to their examination. The study was approved by the Aston University Research Ethics Committee (ID 755) and adhered to the tenants of the Declaration of Helsinki.

### Data collection

**Figure 1.**
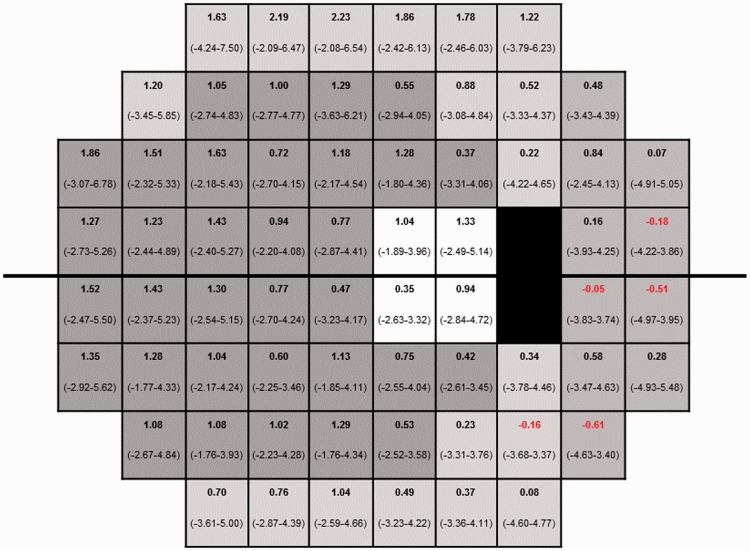
Bias (bold number) and the 95% limits of agreement (unbold numbers) of the threshold values for 66 matching test points between SITA and SPARK. Shading indicates the six regions in the functional map of visual field.^17^ A positive number indicates that the measured threshold was higher for SPARK than for SITA and vice versa.

Following written informed consent, all subjects underwent two visual field assessments on two separate days. At each visit, one randomly selected eye was tested in succession with the Oculus Twinfield 2 (OCULUS Optikgeraete GmbH, Wetzlar, Germany) using the SPARK Precision algorithm and with the Humphrey field analyzer (HFA) (Carl Zeiss Meditec, Dublin, CA, United States) using the 30-2 SITA Standard algorithm. Both instruments use a background luminance of 10 cd/m^2^ and have a maximum stimulus luminance of 10,000 asb. The 30-2 test pattern used in the HFA has a total of 76 test points covering the central 30° field with a square grid of 6° separation.^17^ The SPARK Precision algorithm deploys a similar grid with a total of 66 test points (30° × 24°) with the uppermost and bottommost rows and two points located in the blind spots missing compared to the SITA 30-2 test grid.^10^ For the purpose of this study, all participants were examined with the four phases of the SPARK Precision algorithm. In the first phase, the 66 threshold values corresponding to the 66 test points are estimated by directly examining only six points, one in each functional region.^15^ Thresholds for all other locations are determined by interpolation, but ultimately each of the 66 points is assessed at least once. In the following three phases, the threshold values of the 66-point grid get refined by testing 21 points (per phase) of the six regions (see Figure 1

) and subsequent adjusting of neighbouring points through further interpolation. The final threshold value at each location is determined by calculating the median of the four threshold values derived at each of the four phases.

Participants were given a break of 10 min between the two tests. The order of SITA and SPARK Precision testing was randomised among subjects to minimise learning effects but remained constant between the two visits of a given individual. Consideration of the first and second visits enabled comparison of threshold variability within the SITA and SPARK Precision strategies. The results of the second visit only were used to make a between strategy comparison because they are less influenced by the learning effect.^14,15^

### Data analysis

Test results with poor reliability criteria defined as false positives (FPs) or false negatives (FNs) >20% and fixation losses (FLs) >30% with SITA and FP > 20% and FL > 30% based on SPARK Precision, since it does not measure FN, were excluded from data analysis, as were any fields which exhibited a defect in consecutive visits according to Hodapp–Parrish–Anderson classification of glaucoma.^18^ A comparison of threshold variability within each of the SITA and SPARK Precision strategies was made by carrying out a Bland–Altman analysis for the 66 stimulus locations in SPARK Precision and the 66 stimulus locations in SITA which were coincident with the SPARK Precision test pattern. To make a between strategy comparison, the mean sensitivities of the 66 points test grid of SPARK Precision and matching stimulus locations in the SITA test grid from the second visit were calculated for each patient and a pointwise analysis between strategies was carried out to determine the threshold agreement between the two strategies. The results from the test points of the uppermost and bottommost rows and two points located at the blind spot for SITA were excluded. The central threshold in SPARK Precision was also excluded in the calculation of MS. The left eye results were transposed into right eye format for the purpose of the analysis. The association between the MS and age was determined for each strategy, and test duration of each strategy was recorded and compared between the strategies.

Differences in threshold estimation between visits were completed for each method separately. Differences between the two methods were evaluated using pointwise analysis for each stimulus location (using only the 66 corresponding data points and only visual field (VF) data obtained at visit 2 of each participant). A comparison between the two methods of the six regions according to the functional map described by de la Rosa et al.^10^ was made and additionally in terms of three eccentric test zones defined as central (<10°), mid-peripheral (10°–20°) and peripheral (>20°).

### Statistical analysis

Normality of the threshold data was determined by the Shapiro–Wilk test and the Wilcoxon Signed Rank test which was used for between strategy comparisons. Bland–Altman plots were used to determine the agreement of the mean sensitivities within and between both testing algorithms, and regression testing was conducted to determine proportional bias. All correlations were determined using Spearman’s correlation coefficient. A one-way analysis of variance (ANOVA) was used for comparison among the test zones followed with Tukey’s honestly significant difference (HSD) post hoc analysis. While this constitutes multiple comparisons, we did not apply any correction on the basis of the exploratory nature of these particular analyses as recommended by Armstrong.^19^ Statistical significance was set at a level of p < 0.05.

## Results

### MS

All visual field test results were within our inclusion criteria. Analysing individual error responses however exhibited that all participants of the included VF data exhibited FPs and FLs <20% for both test strategies and FN < 20% for SITA. Absolute values for FPs were 2.1 ± 3.0 (SITA) and 2.7 ± 6.0 (SPARK Precision), FNs are only measured for SITA (0.8 ± 0.5) but not for SPARK Precision. FLs are logged for both test strategies, and absolute values were as follows, SITA: 3.4 ± 3.8 and SPARK Precision: 1.0 ± 3.6. Between strategy analyses were carried out in all 83 subjects. SITA exhibited a significantly lower MS (on average 0.93 dB less than SPARK Precision), Wilcoxon Signed Rank test: Z = −6.118, p < 0.001. The examination with SPARK was, when adjusting for the fact that it uses 10 test locations less than SITA, on average 33% faster to complete than SITA (p < 0.01) (Table 1

**Table 1.** The mean sensitivity and test duration of SITA and SPARK in normal subjects.

	Mean	SD	Median	Range	
				Min	Max
Mean sensitivity (dB)
SITA	30.81	1.32	30.94	27.32	33.61
SPARK	31.74	1.50	31.94	28.06	34.32
Test duration (min)
SITA	6.05	0.67	5.87	4.92	8.18
SPARK	3.50	0.13	3.50	3.23	3.82

SD: standard deviation.

shows the raw data duration times for both methods).

**Figure 2.**
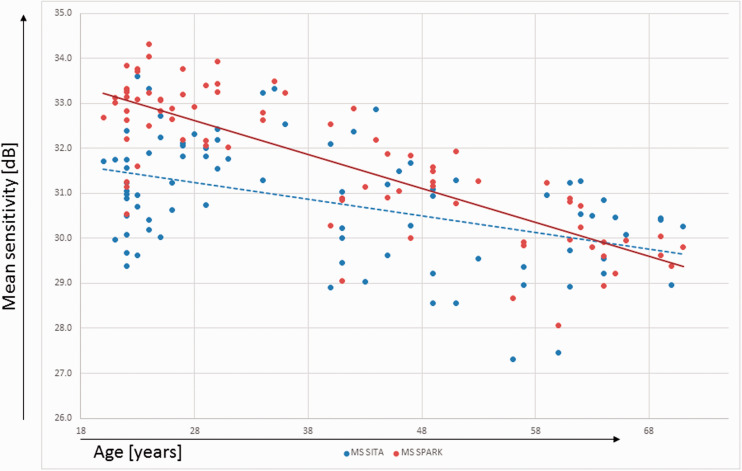
Correlation between age and mean sensitivity or SPARK Precision and SITA.

The mean sensitivities of SITA and SPARK Precision were both shown to be negatively correlated to the subject’s age (Spearman correlation coefficient: rho = −0.382, p < 0.001 for SITA; rho = −0.752, p < 0.001 for SPARK; see Figure 2

).

A positive correlation between group mean sensitivities of SITA and SPARK Precision was found (Spearman correlation coefficient: rho = 0.713, p < 0.001). The bias of the MS difference between SITA and SPARK Precision and the 95% limits of agreement (LoA) was 0.92 dB (LoA: −1.12, 2.97 dB). As 95% of the data points were located within the LoA, further linear regression testing on the differences showed no statistically significant proportional bias (t = 1.713, p = 0.09).

### Pointwise analysis

Fourteen subjects showed poor reliability in the first visual field visit. These subjects were excluded from the within threshold test strategy comparisons which was carried out on 69 subjects (mean age: 37.6 years; SD: 16.0; range: 20–71). Within test strategy comparisons were made by calculating the mean difference in threshold for each visit for a given test algorithm (i.e. sensitivity at visit 2 − sensitivity at visit 1). Table 2

**Table 2.** Mean and standard deviation of pointwise mean differences between-visit using SITA and SPARK.

	Type of strategy	Mean	SE
Pointwise mean difference (dB)	SITA	0.37	0.03
	SPARK	0.04	0.02

SE: standard error.

shows that there was much lower test–retest variability in SPARK Precision compared to SITA. The group MS of SITA at the second visit was significantly higher compared to the first visit (mean of visit 1 = 30.58 dB, mean of visit 2 = 30.96 dB; paired t-test: p = 0.003), although these differences are not clinically significant. No statistically significant difference in group MS was found between visits for the SPARK Precision strategy (median of visit 1 = 32.20 dB, median of visit 2 = 32.18 dB; Wilcoxon Signed Rank test: p = 0.522).

The comparison between the two strategies (69 subjects) for the average of pointwise variations between visits of all 66 test points showed that SITA had statistically significant larger variability (Paired t-test: t = 9.175, df = 65, p < 0.001) compared to using SPARK Precision in normal subjects. Between test strategy comparisons were made by calculating the mean difference between the measured threshold of SPARK Precision and SITA on a pointwise basis (Figure 1). At 92% of the 66 stimulus locations, SPARK Precision recorded a higher sensitivity than SITA.

A statistical difference was found across the six regions (Table 3

**Table 3.** Mean and standard deviation of bias in MS between strategies according to six functional regions described by de la Rosa et al.^17^

Region	Bias in MS (dB)	
	Mean	SD
Superior	1.37	0.69
Superior nasal	1.06	0.37
Inferior nasal	1.10	0.46
Inferior	0.43	0.37
Temporal	0.11	0.46
Central	0.92	0.41

MS: mean sensitivity; SD: standard deviation.

; one-way ANOVA: F = 11.192, p < 0.001), whereas the largest bias of mean sensitivities between SPARK Precision and SITA was found in the superior region. Post hoc analysis using Tukey’s HSD showed that the temporal region had the lowest bias compared to all other regions (p < 0.05) except for the inferior region (p = 0.633). The same analysis in terms of central, mid-peripheral and peripheral rings of eccentricity yielded no statistically significant difference among these three regions (one-way ANOVA: F = 0.146, p = 0.865) (Table 4

**Table 4.** Mean and standard deviation of bias in MS between strategies according to eccentricity.

Location	Bias of MS (dB)	
	Mean	SD
Central (<10°)	0.82	0.32
Mid-peripheral (10°–20°)	0.85	0.49
Peripheral (>20°)	0.91	0.76

MS: mean sensitivity; SD: standard deviation.

).

## Discussion

In the presented sample, the threshold estimates produced by SPARK Precision and SITA were found to have significant associations with age. The mean threshold estimates decreased with age in agreement with previous reports^17,20–24^ by approximately −0.4 and −0.8 dB/decade for SITA and SPARK Precision, respectively, which is close to the rates reported by others.^17,22,25,26^

The MS of SPARK Precision was statistically but not clinically higher than that of SITA (0.92 dB). Bland–Altman analysis showed a high level of agreement between the two strategies in this group of normal subjects. According to the evaluation criteria suggested by Luithardt et al.,^27^ a bias of less than 1 dB indicates a good agreement. However, LoA greater than 4 dB can be classed as clinically acceptable. The most likely explanations for the higher threshold estimates produced by SPARK Precision are first reduced fatigue and second that threshold responses in SPARK Precision are interpolated from acquired thresholds at limited stimulus locations whereas each stimulus location in SITA is derived from independent assays at each stimulus location and therefore is influenced more by subject variability. The test duration of SITA, which uses 10 more test locations than SPARK, was on average 6 min compared with 3.5 min in SPARK Precision. While this time reduction is significant, it is not an absolute time reduction as SPARK Precision tests only 66 points whereas SITA is assessing 76 points. Nevertheless, any reduction in testing time which does not sacrifice accuracy is desirable to avoid fatigue, in particular when testing older subjects. A reduction in MS at a rate of 0.08–0.1 dB has been attributed to the fatigue effect.^6^ Thus, the faster examination time of SPARK Precision could partly explain the higher group MS. SITA utilises two likelihood functions developed from prior knowledge of normal and glaucomatous models which continue to be adjusted according to the patients’ response during the test.^8^ The final threshold estimate of SITA is derived according to the pre-determined precision in the mathematical model selected (Standard or Fast). Whereas SPARK Precision uses the first phase stimulus responses to determine the threshold estimates for the other three phases, in these phases, the threshold estimates appear to be predominantly determined by interpolation and multiple regression equations which could be considered as a smoothing of the acquired data, reducing threshold variability. This smoothing effect can lead to reduced accuracy in detecting more sharply demarked areas of VF loss as well as a lack of precision in evaluating the depth of the VF loss. As SPARK Precision can be seen a logical progression from the TOP algorithm, it has distinct differences to its ‘predecessor’. While SPARK Precision has built on the basic ideas of TOP which are to utilise the correlation between neighbouring locations and is carrying out the test in four phases, there are distinct differences between the two test methods. TOP has been shown to lack accuracy in regards to estimating the spatial extent and absolute sensitivity loss of VF defects.^28^ While both employ four phases to generate their threshold values, TOP measures fewer points than SPARK Precision which presents stimuli at each test location at least once. This may appear as a relatively small difference between methods but has a significant impact on the accuracy of the final thresholds obtained. This is because if a patient does not respond to a stimulus presented in TOP which could be detected, then all its neighbouring points will be influenced by this, in SPARK Precision, where each test location is at least measured once, the effect of such an error is less pronounced.

Pointwise comparison of group mean sensitivities between strategies showed that most of the test points had higher threshold estimate when using SPARK Precision compared to SITA. A higher sensitivity using SITA was only present at five stimulus locations in the temporal field. This region also yielded the lowest mean bias when assessing the six regions described by de la Rosa et al.,^29^ but we are unable to provide an explanation for this finding. In conclusion, the clinical examination of normal subjects using SPARK Precision yields a sensitivity profile across the visual field which is similar to SITA Standard but in a relatively faster examination time. Our test results are not generalizable in that the clinical validity of SPARK Precision in patients with a variety of systemic and ocular conditions characterised by visual field loss will need to be assessed in a separate trial including patients with established visual field defects.
